# Two Monthly Continuous Dynamic Model Based on Nash Bargaining Theory for Conflict Resolution in Reservoir System

**DOI:** 10.1371/journal.pone.0143198

**Published:** 2015-12-07

**Authors:** Mehran Homayounfar, Mehdi Zomorodian, Christopher J. Martinez, Sai Hin Lai

**Affiliations:** 1 Department of Civil Engineering, Faculty of Engineering Building, University of Malaya, Kuala Lumpur, Malaysia; 2 Associate Professor, Department of Agricultural and Biological Engineering, University of Florida, Gainesville, Florida, United States of America; 3 Associate Professor, Department of Civil Engineering, Faculty of Engineering Building, University of Malaya, Kuala Lumpur, Malaysia; Southwest University, CHINA

## Abstract

So far many optimization models based on Nash Bargaining Theory associated with reservoir operation have been developed. Most of them have aimed to provide practical and efficient solutions for water allocation in order to alleviate conflicts among water users. These models can be discussed from two viewpoints: (i) having a discrete nature; and (ii) working on an annual basis. Although discrete dynamic game models provide appropriate reservoir operator policies, their discretization of variables increases the run time and causes dimensionality problems. In this study, two monthly based non-discrete optimization models based on the Nash Bargaining Solution are developed for a reservoir system. In the first model, based on constrained state formulation, the first and second moments (mean and variance) of the state variable (water level in the reservoir) is calculated. Using moment equations as the constraint, the long-term utility of the reservoir manager and water users are optimized. The second model is a dynamic approach structured based on continuous state Markov decision models. The corresponding solution based on the collocation method is structured for a reservoir system. In this model, the reward function is defined based on the Nash Bargaining Solution. Indeed, it is used to yield equilibrium in every proper sub-game, thereby satisfying the Markov perfect equilibrium. Both approaches are applicable for water allocation in arid and semi-arid regions. A case study was carried out at the Zayandeh-Rud river basin located in central Iran to identify the effectiveness of the presented methods. The results are compared with the results of an annual form of dynamic game, a classical stochastic dynamic programming model (e.g. Bayesian Stochastic Dynamic Programming model, BSDP), and a discrete stochastic dynamic game model (PSDNG). By comparing the results of alternative methods, it is shown that both models are capable of tackling conflict issues in water allocation in situations of water scarcity properly. Also, comparing the annual dynamic game models, the presented models result in superior results in practice. Furthermore, unlike discrete dynamic game models, the presented models can significantly reduce the runtime thereby avoiding dimensionality problems.

## Introduction

Inefficiency of classical conflict resolution techniques [1–6] in dealing with conflicting issues in water allocation among different users have drawn attention to utilizing innovative alternatives for resolving conflict. A number of approaches such as Nonlinear Programing [7, 8], Compromise Programming [9–11], Analytic Hierarchy Process [12, 13] and Fuzzy Set Analysis [14–16] have been utilized to deal with conflict situations.

Game theory as another approach for conflict resolution has been broadly applied in water resource management [17–25]. The applicability of Game Theory to water resources management and conflict resolution was reviewed by Carraro et al. [26] and Madani [27] through a series of non-cooperative water resource games. Similar studies have been conducted by Parrachino et al. [28] and Zara et al. [29], evaluating the application of cooperative game theory to water resources and environmental issues. In another study by Madani and Dinar [30], a number of cooperative game theoretic solutions (i.e. the core, Nash-Harsanyi, Shapley, and nucleolus) are formulated and applied through a numerical groundwater example. Considering applied cooperative solutions, they evaluate how common pool resources users share the gains obtained from cooperation efficiently and fairly.

In the context of game theory, Nash bargaining is a typical game introduced by Nash [31] applicable to model conflict situations. Subsequently, a Nash Bargaining Solution (NBS) is a solution (i.e. Pareto efficient solution) to this game. Harsanyi [32] developed an equilibrium solution for an n-person bargaining problem based on an initial Nash equilibrium solution for the two-player case. According to Thomson [33], Nash’s solution is generally accepted as the typical framework for bargaining problems. So far, many researchers have utilized NBS to deal with conflict situations in different water-related problems. In the area of water quality NBS has been utilized in a number of studies [34–36]. Regarding optimal reservoir management also NBS has been used for conflict resolution [37–40]. Karamouz et al. [3] used Nash product for formulation of the objective function of a reservoir water allocation model and used resiliency and vulnerability indices to evaluate the performance of optimization algorithms. Results showed the significance of the application of conflict resolution models, such as the Nash theory in the regional scale especially in complicated water supply systems.

A number of discrete dynamic models based on Nash Bargaining Theory have been extended to provide an original way to overcome problems by taking into account the interaction between different objectives, behaviors and preferences of water users. Kerachian and Karamouz [35] created a stochastic dynamic model for conflict resolution in reservoirs and river basins. The objective function was defined based on the expected value of the Nash product. Another attempt based on the same approach was conducted by Ganji et al. [17, 18, 41] to develop a discrete stochastic dynamic model to simulate the competition between water users downstream of a reservoir. All these models are powerful in dealing with a dynamic game problem but suffer the setback of high computational effort in getting appropriate solutions [42]. Additionally, in the same context, most of the recent continuous approaches are known as powerful tools for generating operating policies. However, many of the developed models have been structured annually. Compared to the models which work on a monthly basis, this can lead to inefficiencies in reservoir operation.

In this study two continuous dynamic optimization models are developed using Nash bargaining theory. The principal aim is to introduce monthly continuous dynamic approaches applicable to single reservoir systems in order to maximize long-term utility associated with reservoir management and water users. Due to the continuous form of the state variable, these approaches will not suffer from the curse of dimensionality. Also they result in practical operating policies compared to annual dynamic structures. The first model employing constrained state formulation introduced previously by Fletcher and Ponnambalam, [43], provides an optimization approach for water allocation in arid and semi-arid regions. The objective function is structured based on NBS and optimized subject to the statistical moments (The first and second moments) of the storage state variable of a reservoir system. The second model is a dynamic approach structured based on continuous state Markov decision models. We employ the collocation method to solve this model. The presented model was developed based on a previous study by Homayounfar et al. [44]. The presented model results in monthly operating policies distributing limited available water among different users using NBS. The Zayandeh-Rud reservoir system in central Iran is selected to demonstrate the capabilities of the presented models in comparison with the results of the annual form of the dynamic game model, (i.e. second presented model) [42], a discrete stochastic dynamic game [18], and a Bayesian Stochastic Dynamic Programming (BSDP) [45] model of reservoir operation.

As an outline of the present research, first, the theory of Constricted state formulation is presented and the objective function and corresponding constraints of the dynamic model (first model) created based on this theory are introduced. Afterwards, the Markov decision Process is briefly presented as a dynamic solution to deal with conflict in water allocation in reservoir system. Then, the theoretical framework of the second model and corresponding solution (collocation method) is presented. The model adjustment for monthly use and the model structure associated with the second model will be explained as the final part of the methodology section.

## Methodology

### Optimization model for the dynamic of single reservoir

#### Theory of Constricted state formulation

Fletcher and Ponnambalam, [43] introduced a stochastic optimization model to provide an efficient management in reservoir system operation with an acceptable level of reliability. The structure of the aforementioned method was similar to Stochastic Dynamic Programming. However, the state variables (reservoir storage) were non-discrete, thereby a continuous dynamic optimization method was formed. Statistical parameters, the first and second moment of the storage variable (mean and variance of the reservoir storage) play important roles in characterizing the probability distribution of the storage variable. Using these parameters as exerted constraints in the model not only provides an explicit consideration to the bounded storage variable in the reservoir system but also the model would not involve any discretization of the system variables.

Regarding the preceding study conducted by Fletcher and Ponnambalam, [43], the first and second moments of the storage stage are calculated and taken into account as constraints for an optimization problem. Considering the continuity equation for the reservoir system, Eq 1, and the indicator function, Eq 2, the first and second moments of the storage state variable, Eq 3 and Eq 4 respectively, are derived.

$$s_{t}=s_{t-1}+(I_{t}+\eta_{I}^{t})-R_{t}$$ (1)

$$1_{\left[s_{\min(t),}s_{\max(t)}\right]}(\overset{\wedge }{s_{t}})=\left\{ \begin{array}{l} 1\;for\;s_{\min(t)}\leq \overset{\wedge }{s_{t}}\leq s_{\max(t)}\\ 0\;otherwise \end{array}\right.$$ (2)

Where *s*_*t*_ and *s*_*t-1*_ represent the water storage level in reservoir at time t and t-1 respectively. *R*_*t*_ is the water release decision, *I*_*t*_ is the long term average of monthly inflow into reservoir, *s*_*min(t)*_ and *s*_*max(t)*_ are, respectively, minimum and maximum amount of water storage in reservoir and *ƞ*_*I*_^*t*^ is the random component associated with the inflow at time t.

$$\begin{array}{l} E(s_{t})=E(s_{t-1})+\left\{\begin{array}{l} \frac{1}{2}(-erf(-\frac{\left(s_{\max(t)}-(I_{t}-R_{t})-E(s_{t-1})\right)}{2{\left(Var(\eta_{I}^{t})\right)}^{1/2}})\\ -erf(-\frac{\left(s_{\min(t)}-(I_{t}-R_{t})-E(s_{t-1})\right)}{2{\left(Var(\eta_{I}^{t})\right)}^{1/2}})) \end{array}\right\}\;\left[I_{t}-R_{t}\right]\\ \;+\left\{\frac{1}{2}(1+erf(-\frac{\left(s_{\max(t)}-(I_{t}-R_{t})-E(s_{t-1})\right)}{2{\left(Var(\eta_{I}^{t})\right)}^{1/2}}))\right\}\;\left[s_{\max(t)}-E(s_{t-1})\right]\\ \;+\left\{\frac{1}{2}(1-erf(-\frac{\left(s_{\min(t)}-(I_{t}-R_{t})-E(s_{t-1})\right)}{2{\left(Var(\eta_{I}^{t})\right)}^{1/2}}))\right\}\;\left[s_{\min(t)}-E(s_{t-1})\right] \end{array}$$ (3)

$$\begin{array}{l} E{\left(s_{t}\right)}^{2}=E{\left(s_{t-1}\right)}^{2}+\left\{\begin{array}{l} \frac{1}{2}(-erf(-\frac{\left(s_{\max(t)}-(I_{t}-R_{t})-E(s_{t-1})\right)}{2{\left(Var(\eta_{I}^{t})\right)}^{1/2}})\\ -erf(-\frac{\left(s_{\min(t)}-(I_{t}-R_{t})-E(s_{t-1})\right)}{2{\left(Var(\eta_{I}^{t})\right)}^{1/2}})) \end{array}\right\}\\ \;*\left[{\left(I_{t}-R_{t}\right)}^{2}+Var(\eta_{I}^{t})+2E(s_{t-1})*(I_{t}-R_{t})\right]\\ \;\left\{\frac{1}{2}(1+erf(-\frac{\left(s_{\max(t)}-(I_{t}-R_{t})-E(s_{t-1})\right)}{2{\left(Var(\eta_{I}^{t})\right)}^{1/2}}))\right\}\;\left[s_{\max(t)}-E(s_{t-1})\right]\\ \;+\left\{\frac{1}{2}(1-erf(-\frac{\left(s_{\min(t)}-(I_{t}-R_{t})-E(s_{t-1})\right)}{2{\left(Var(\eta_{I}^{t})\right)}^{1/2}}))\right\}\;\left[s_{\min(t)}-E(s_{t-1})\right] \end{array}$$ (4)

The symbols *erf()* and *Var()* are, respectively, the error function and variance and illustrated as follows:

$$erf(x)=\frac{2}{\sqrt{\pi }}\int_{0}^{x}e^{-t^{2}}dt$$ (5)

$$Var(x)=\frac{\sum_{i=1}^{n}{\left(x_{i}-\bar{x}\right)}^{2}}{n}$$ (6)

In the Eq 3 and Eq 4, *E(s*_*t*_ *)* and *E(s*_*t*_ *)* ^*2*^ are the first moment (Mean) and second moment (Variance) of the storage variable. Further detailed explanation regarding the extension of the first and second moment equations and constrained state formulation is available in studies done by Fletcher and Ponnambalam [43].

#### Objective Function of the continuous optimization model

In this method, the long-term variation of the state variable are imposed on the optimization model through constraints stated by Eq 3 and Eq 4. Therefore, the optimal value of long-term utilities corresponding to water users and reservoir management are achieved from the optimization. Since the objective function should have a conflict resolution structure, we defined the function based on the Nash product. In order to maximize the long-term average of the Nash product function, the first moment of Nash function is calculated. (Eq 7).

$$Objective\;function=E(Ln(U(s_{t}))+\sum_{i=1}^{n}Ln(U(x_{i}))$$ (7)

where, *E()* is the expectation of the function and *Ln()* is the natural logarithm. Also *x*_*i*_ and *U(x*_*i*_ *)* are respectively allocated water to the water user *i* and the corresponding utility functions. St and *U(s*_*t*_ *)* are the reservoir storage in state *t* and the corresponding utility function. In order to estimate Eq 5 the ninth order Taylor series approximation is used.

#### Constraints on the continuous dynamic model

Constraint of the minimum and maximum allocated water to each sector (Eq 8)

$$0\leq x_{i}\leq x_{i,\max}$$ (8)

where *x*_*i*_ and *x*_*i*,*max*_ are respectively allocated water and maximum allocated water to each sector.

- Constraint of the maximum and minimum total released water.

$$R_{\min}\leq R\leq R_{\max}$$ (9)

where *R*_*min*_ and *R*_*max*_ are, respectively, minimum and maximum allowed release water and *R* is the total monthly release water.

- Constraint of total available water

$$\sum_{i=1}^{n}x_{i}\leq R$$ (10)

- Constraints of the first and second moments related to the first and last months of the year.
  These constraints guarantee that reservoir condition at the beginning of each year is similar to the end of the previous year.

$$E(s_{1})=E(s_{13})$$ (11)

$$E{\left(s_{1}\right)}^{2}=E{\left(s_{13}\right)}^{2}$$ (12)

where in Eq 11 and Eq 12, *s*_1_ and *s*_13_ are, respectively, reservoir storages in the last month and first month of every two consecutive year.

- The Eq 3 and Eq 4 are also contributed into constraints as well.

### Continuous Dynamic Optimization model and corresponding collocation solution

#### Dynamic view of conflict in water allocation in reservoir system

Problems dealing with conflict in water consumption among different users based on experts’ views can be defined as both static and dynamic cases. Even though applying a static frame for a problem makes it easier to solve, the dynamic nature of the problem will be ignored. In this content and regarding conflict in reservoir operation and water allocation, the sequential nature of the reservoir management decisions, together with the inherent randomness of natural water inflows explain the frequent modeling of reservoir management problems as Markov decision processes and their optimization by stochastic dynamic programing.

When an allocation problem along an infinite time horizon is under discussion, the life time utility function can be presented by Eq 13. The main goal is to maximize the overall water users’ utilities (*U*_*s*_). In this equation, vectors *x = {x*_*i*_, *i = 1…*, *n}* and *U(x*_*i*_ *)* denote allocated water to different water users and the utilities produced by using the allocated water, respectively.

$$V_{t}\left(S_{t},\varepsilon_{t}\right)=Max_{x_{t}^{1},\ldots ,x_{t}^{n}},\{U_{s}\left(x_{t}^{1},\ldots ,x_{t}^{n},R_{t};t\right)+\gamma E[V_{t+1}\left(g\left(S_{t},I_{t},\varepsilon_{t}\right)\right)]\}$$ (13)

where: *t* shows the time step of the system, *S*_*t*_ and *R*_*t*_ are the reservoir storage and water release at time *t* and *I*_*t*_ is the inflow. Moreover, *γ* is the discount factor and *V*_*t*_ *(S*_*t*_ *)* states the indirect utility function (Value Function) at time *t*. On the right side, *g(S*_*t*_, *I*_*t*_, *ɛ*_*t*_ *)* states the transition equation (Eq 14) and *ε*_*t*_ is the factor by which the randomness associated with inflow discharge is imposed and is called exogenous random shock (this parameter is the same as *ƞ*_*I*_^*t*^ being already defined in section (2.2.1)). Also *E()* denotes the expectation of the value function.

$$g\left(S_{t},I_{t},\varepsilon_{t}\right)\equiv S_{t+1}=S_{t}+\left(I_{t}+\varepsilon_{t}\right)-R_{t}$$ (14)

Eq 13 is known as the Bellman equation. The first component in this equation represents the utility derived from immediate consumption at any given time whereas the second component represents the value of optimal lifetime consumption starting one period from state *t*.

In this context, the multiplicative form of utility functions of water users (*U*_*x*_ *(x*_*t*_^*i*^ *)*) and reservoir operator (*U*_*s*_ *(S*_*t*_ *)*), which is known as the NBS, is considered as the intermediate objective function (Eq 15).

$$Intermediate\;Objective\;Function=\prod_{x=1}^{n}\left(U_{x,t}-d_{x}\right)*\left(U_{s,t}-d_{s}\right)$$ (15)

In this equation, *d*_*x*_ and *d*_*s*_ represent minimum allocated water to water users and minimum water level in the reservoir, respectively. The main aim is to maximize this product in every proper sub-game over the planning horizon.

#### Collocation method for continuous dynamic model

The value function can be written as a linear combination of a set of *m* linearly independent basis functions *(φ*_*1*_, *φ*_*2*_, *φ*_*3*_,*…*, *φ*_*j*_ *)* associated with *S*_*t*_ (storage as the state variable) and unknown basis coefficients *(c*_*1*_, *c*_*2*_,*…*, *c*_*j*_ *)*:

$$V_{t}\left(S_{t},\varepsilon_{t}\right)\approx \sum_{j=1}^{m}c_{j}\varphi_{j}\left(S_{t}\right)$$ (16)

Considering the collocation method, many interpolation schemes can be employed to approximate the functional equation. Here, one general and practical scheme, namely Chebychey Polynomial and approximation is applied to approximate the functional equation [46].

#### Chebychev approximation function

Chebychev polynomials are associated with a family of orthogonal polynomials described by Judd [47]. Eq 17 is to normalize the domain to the interval [–1, 1], with the Chebychev polynomials defined as in Eq 18:

$$z=2*\left(S_{t}-S_{min}\right)/\left(S_{max}-S_{min}\right)-1$$ (17)

$$\varphi_{j}(S_{t})=T_{j-1}(z)$$

where,

$$\begin{array}{l} T_{0}\left(z\right)=1\\ T_{1}\left(z\right)=z\\ \begin{array}{l} T_{2}\left(z\right)=2z^{2}-1\\ T_{3}\left(z\right)=4z^{3}-3z\\ \begin{array}{l} \;\vdots \\ T_{j}\left(z\right)=2zT_{j-1}\left(z\right)-T_{j-2}\left(z\right) \end{array} \end{array} \end{array}$$ (18)

The linear combination of *m* nonlinear equations and *m* unknowns (Eq 15) can be substituted for the value function in Eq 13. This replacement results in Eq 19:

$$\sum_{j=1}^{m}c_{j}\varphi_{j}\left(S_{t}\right)=Max_{x_{t}^{1},\ldots ,x_{t}^{n}},\left\{U_{s}\left(x_{t}^{1},\ldots ,x_{t}^{n},R_{t};t\right)+\gamma E\left[\sum_{j=1}^{m}c_{j}\varphi_{j}\left(g\left(S_{t},I_{t},\varepsilon_{t}\right)\right)\right]\right\}$$ (19)

To compute this optimization problem the Envelope Theorem can be applied [48, 49].

Miranda and Fackler [46] stated that the policy function is associated with the mean of the random variable in the polynomial optimal control model. Accordingly, in order to compute the expectation term practically in Eq 10, the mean of the random variable (*ε*_*t*_) is taken into account in the state transition function. As a result, the transition equation associated with the stochastic problem turns into the form used in the deterministic model and consequently can be solved similarly.

There are a number of numerical solution methods to cope with nonlinear equation problems such as Newton and quasi-Newton methods [50–52]. In this study, we have used the Newton method to solve this problem.

#### Model adjustment: way to introduce a monthly structure

Using the collocation method to solve Eq 19 results in annual operating policies for a reservoir system and does not evaluate changes in water level in reservoir during the year. In continuous frameworks, the reward functions, *U*_*s*_ *(x*_*t*_^*1*^, *…*,*x*_*t*_^*n*^, *R*_*t*_ *; t)*, associated with different months change over the course of the year. Therefore, the second part of the right-hand side, $\gamma \sum_{k=1}^{l}\left[\sum_{j=1}^{m}{w_{k}c}_{j}\varphi_{j}(g(S_{t},I_{t},\varepsilon_{t}))\right]$, cannot be used as an approximation for the rest of the year in its current form. To overcome this issue, the second part of the Eq 19 is modified and divided into two parts, as shown in Eq 19

$$\sum_{Mi=M}^{12}\left[\overset{l}{\underset{k=1}{\sum }}{\left[\sum_{j=1}^{m}w_{k}c_{j}\varphi_{j}\left(g\left(S_{t},I_{t},\varepsilon_{t}\right)\right)\right]}_{Mi}\right]=\overset{l}{\underset{k=1}{\sum }}{\left[\sum_{j=1}^{m}w_{k}c_{j}\varphi_{j}\left(g\left(S_{t},I_{t},\varepsilon_{t}\right)\right)\right]}_{Mi=M}+\sum_{Mi=M+1}^{12}\left[\overset{l}{\underset{k=1}{\sum }}\left[\sum_{j=1}^{m}w_{k}c_{j}\varphi_{j}\left(g\left(S_{t},I_{t},\varepsilon_{t}\right)\right)\right]\right]$$ (20)

In Eq 20, the left-hand side shows the optimal approximation of value functions for the time interval starting from state *t+1* (M) up to the end of the year. On the right-hand side, the first part is the value function approximation related to state *t+1* while the second part represents the value function approximation from state *t+2* to the end of the year. Combining Eq 20 and Eq 19 results in Eq 21:

$$\sum_{j=1}^{m}c_{j}\varphi_{j}\left(S_{t}\right)=Max_{x_{t}^{1},\ldots ,x_{t}^{n}}\left\{U_{s}\left(x_{t}^{1},\ldots ,x_{t}^{n},R_{t};t\right)+\overset{l}{\underset{k=1}{\sum }}\left[\sum_{j=1}^{m}w_{k}c_{j}\varphi_{j}\left(g\left(S_{t},I_{t},\varepsilon_{t}\right)\right)\right]+\sum_{Mi=M+1}^{12}\left[\overset{l}{\underset{k=1}{\sum }}\left[\sum_{j=1}^{m}w_{k}c_{j}\varphi_{j}\left(g\left(S_{t},I_{t},\varepsilon_{t}\right)\right)\right]\right]\right\}$$ (21)

#### Model Structure

**Fig 1:**
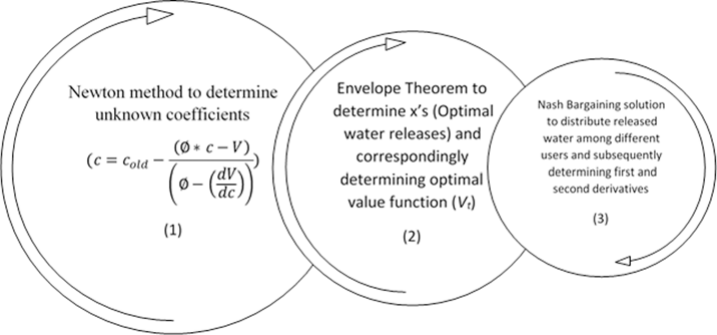
Sequential steps to solve Eq 19.

**Fig 2:**
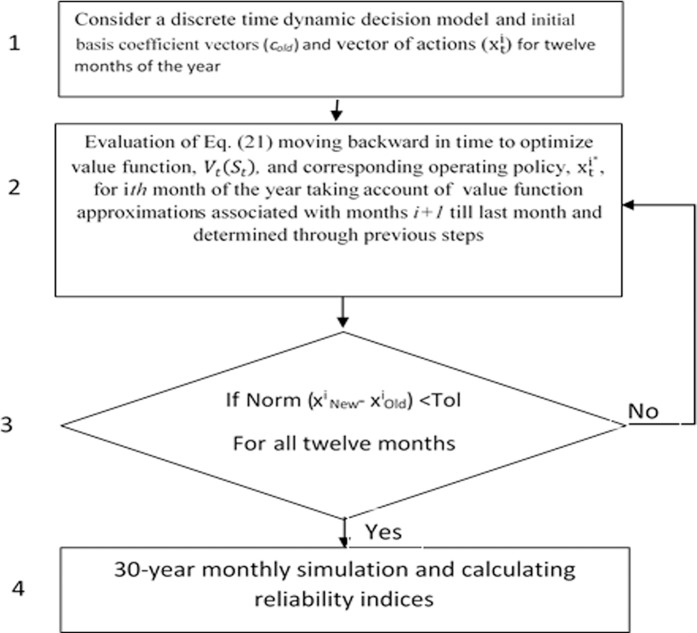
Illustration of solving Eq 21 for each month along the year moving backward.

In order to describe structure of the model, considering Fig 1, we first illustrate the steps to solve Eq 19 annually. Then, the algorithm with which different months will be connected together will be explained by Fig 2.

Fig 1 provides an illustration on the sequential steps to determine the aforementioned values (i.e. optimal value function and optimal operating policy) annually. The model is comprised of three interconnected cycles to determine the share of different water users, optimal water releases (x’s) and unknown coefficients (c’s) respectively. For this purpose, taking initial unknown coefficients into account, the optimal water releases are calculated and used in a recursive algorithm to update unknown coefficients (cycles 1 and 2, Fig 1). Meanwhile, to determine the optimal water releases by means of the Envelope Theorem, we need to know water shares associated with every user. Therefore, employing NBS the corresponding values (i.e. allocated water share to every user) are determined (cycle 3, Fig 1) and used to update x’s and determining optimal water releases in every trial.

Fig 2 shows the algorithm with which different months will be connected. As the first step, Eq 21 is solved for the last month of the year (December) thereby determining the optimal value function, *V*_*t*_ *(S*_*t*_ *)*, and corresponding operating policy, x_t_^i^* associated with December. Then, moving backward, the same parameters are calculated for other months of the year (Step 4, Fig 2) taking into account of value function approximations associated with later months and determined in previous steps. This procedure is continued up to the point when optimal water allocation policies associated with each month remain constant for two subsequent iterations. For example, allocation policies associated with the third month of year (March) calculated through sequential steps 22 and step 10. For this purpose, a predefined small value of tolerance is considered (see Tol in Fig 2).

Applying this approach makes it possible to use Eq 19 in reservoir system operation on a monthly basis and determine the optimal value function, *V*_*t*_ *(S*_*t*_ *)*, and corresponding optimal operating policy, x_t_^i^*, for each month of the year.

## Results and Discussion

### Case Study

**Fig 3:**
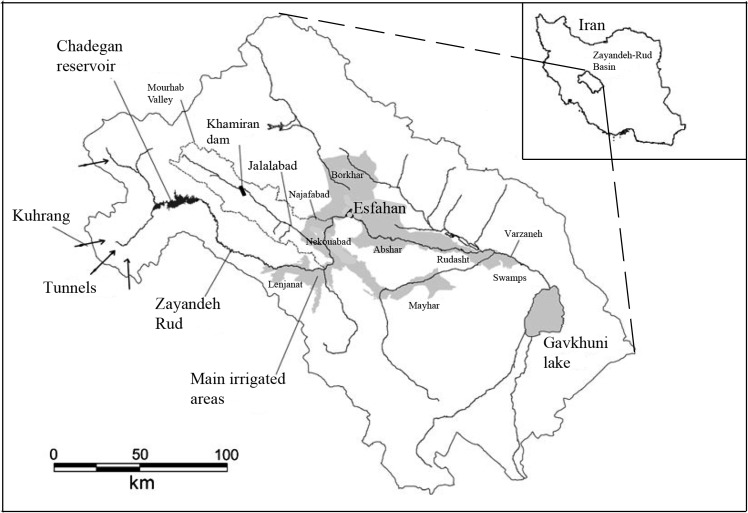
Zayandeh-Rud river basin and reservoir location.

In this study, the Zayandeh-Rud river basin (Fig 3), which is one of the major river basins in Iran with a catchment area of 4,200 square kilometers, is used as a case study to examine the proposed conflict resolution method. The Zayandeh-Rud reservoir with an effective capacity of 1,250 million cubic meters located at the upstream of the river basin provides water for approximately three million people who live in the city of Isfahan and surrounding suburb areas. In addition, the reservoir supplies water for the domestic, industrial and agricultural sectors as well. Apart from that, the reservoir is important in providing water for hydropower, recreational use, and in stream flow. The great demand from various sectors exerts pressure on the river basin especially during summer when the river basin fails to meet the demand. In addition, the effects of climate change have made this problem even worse in the recent decade.

A great deal of research has been conducted on the Zayandeh-Rud basin. Zahraie and Hosseini [53], developed an optimization model based on a genetic algorithm (GA) considering variations in water demands. The efficiency of their proposed model was assessed by performing a long-term simulation of the Zayandeh-Rud reservoir. Madani and Marino [54], applied a system dynamic framework to develop the Zayandeh-Rud Watershed Management and Sustainability Model (ZRW-MSM). Homayounfar et al. [44], developed an annual non-discrete stochastic dynamic game model for reservoir operation and its corresponding solution was based on the collocation method. In this study, the logarithms of the 30-year monthly inflows to the reservoir were modeled with the Thomas-Fiering model. The model reproduces the mean and variance of flows in each of the months and the month to month correlation of the flows. Using these statistical parameters, stream flows are reproduced for every month along the year and utilized for reservoir optimization. The model was structured based on two water user groups (the agriculture and the miscellaneous group which consists of industrial, domestic and environmental sectors), plus the reservoir operator.

### Utility function development

The utility data of water users and the resource manager (reservoir operator) was obtained via previous research conducted by Ganji et al. [17, 18].

The water allocated to different water users is shown by x_t_ = {x_t_^i^, I = 1, 2} Subsequently, *U*_*x*_ *(x*_*t*_^*i*^ *)* and *U*_*s*_ *(S*_*t*_ *)* denote the utilities associated with various users and the reservoir operator, respectively. Utility functions are structured based on quadratic equations. Table 1 presents the coefficients of the quadratic equations of utility values (a*, b* and c*) applied in Eq 22 for both users (agriculture sector and other sectors). Eq 23 also shows the quadratic statement applied for the reservoir operator. The value of a utility function reaches the maximum 1 (100%) when the volume of allocated water *(x*_*t*_^*i*^ *)* comes to the maximum water requirement. Conversely, the value of utility hit the lowest level (0.0%) when the vector *(x*_*t*_^*i*^, *i = 1*, *2)* reaches the boundaries as presented in Table 2. Moreover, the value given to the outside of the boundaries is zero.

$$\{\begin{array}{c} U_{x}\left(x_{t}^{i}\right)=a^{*}{x_{t}^{i}}^{2}+b^{*}x_{t}^{i}+c^{*}\;x_{Max}^{i}\geq x_{t}^{i}\geq x_{Min}^{i}\\ \;0\;Otherwise \end{array}$$ (22)

$$\{\begin{array}{c} U_{s}(S_{t})=3.25{\left(S_{t}\right)}^{2}+3.41\left(S_{t}\right)+0.005\;1400\geq S_{t}\geq 150,\\ \;0\;Otherwise \end{array}$$ (23)

**Table 1:** The coefficients of the quadratic equations of utility values for different water users.

Month	Other users**			Agriculture user		
	a*	b*	c*	a*	b*	c*
January	-0.00834	0.470401	-5.63305	-0.11097	0.485445	0.460016
February	-0.00963	0.564644	-7.27352	-0.11099	0.485437	0.460192
March	-0.01121	0.674174	-9.13224	-0.11095	0.48545	0.46001
April	-0.01098	0.657874	-8.85444	-0.00059	0.08375	-1.98355
May	-0.01427	0.893245	-12.9731	-6E-05	0.02685	-1.98405
June	-0.02023	1.323503	-20.7112	-6.6E-06	0.007606	-1.17447
July	-0.02353	1.574739	-25.4847	-1.7E-05	0.014329	-1.98011
August	-0.02023	1.323503	-20.7112	-2.4E-05	0.016998	-1.98823
September	-0.02023	1.323503	-20.7112	-7.8E-05	0.03055	-1.98349
October	-0.01522	0.94553	-13.6817	-0.00027	0.056998	-1.98352
November	-0.01213	0.729813	-9.97622	-0.00739	0.297055	-1.98606
December	-0.00961	0.556742	-7.05902	-0.11098	1.151331	-1.98514

(a*, b* and c*): the coefficients of the quadratic equations of utility values for different water users.

** Including: Domestic, Industrial and Environmental users

**Table 2:** The given allocated water which results in the maximum and minimum values of the utility function for different users (MCM).

Month	Agriculture user		Other users*	
	$x_{Min}^{j}$	$x_{Max}^{j}$	$x_{Min}^{j}$	$x_{Max}^{j}$
January	0	2. 1	17.26	27.9
February	0	2.05	19.116	29
March	0	2.1	20.62	30.15
April	30.01	71.4	20.416	30
May	93.61	224.5	22.916	31.5
June	184.01	555	25.916	32.5
July	175.01	420	27.416	33.5
August	148.21	350	25.916	32.5
September	82.241	195	25.916	32.5
October	44.08	105	22.95	31
November	8.47	20	21	30.2
December	2.185	5.1	18.76	28.5

* Including: Domestic, Industrial and Environmental users

### Reliability Indices

In this study, reliability is the key deciding point to evaluate the performance of the model based on the decisions by the reservoir operator and water users. The volumetric reliabilities of the reservoir system is utilized to evaluate the capability and efficiency of the proposed model. For water allocation, the volumetric reliability of the reservoir system, called total reliability, is:

$$R_{v-all}^{T}=\frac{100}{nyear}\sum_{i=1}^{nyear}\left(\frac{Yearly\;supplied\;water}{Yearly\;demand}\right)$$ (24)

where, *nyear* shows the length of the planning horizon. Evaluation of the shortfall and the overflow in the reservoir can be carried out by calculating the reservoir volumetric reliability index (Eq 25):

$$R_{v-st}=\left(1-\frac{Total\;storage\;shortfall\;or\;overflow}{Total\;available\;water\;into\;the\;reservoir\;system\;during\;the\;planning\;horizon}\right)$$ (25)

### Solving the optimization model based on constrained state formulation

In this part, as explained previously in the methodology section, applying the Constrained State formulation (introduced by [43]) and based on Nash Bargaining Theory an optimization model for conflict resolution in a limited water situation is presented.

Comparing the discrete dynamic optimization models, the principal feature of this model is to consider the state variable (water level in the reservoir) as a continuous variable thereby avoiding dimensionality problems and long runtime. In this model, the first and second moments of the storage variable, important to characterizing the probability distribution function of the storage variable, are determined and taken into account as constraints for an optimization problem.

The model run for two water users comprised of agriculture sector and other sectors consisting of industrial sector, domestic sector and environmental sector. Solving the model results in determining the first moment of the state variable (monthly long-term average of water storage in the reservoir), the second moment of the state variable (the variance of the changes in the water storage in the reservoir), and the monthly release of water from the reservoir and the water share for each water user (Table 3). The objective function is represented using Eq 7. The model converged after 389 iterations by trial and error.

**Table 3:** The optimized variables resulted from the continuous optimization model based on constrained state formulation.

Optimized variables	Jan	Feb	Mar	Apr	May	Jun	Jul	Aug	Sep	Oct	Nov	Dec
First moment of the state variable (MCM)	789	842	949	1208	1401	1400	1033	815	731	687	706	747
Second moment of the state variable (*100)	7512	7512	7512	7512	7511	7511	7512	7512	7512	7512	7512	7512
Water release from reservoir (MCM)	22	23	24	58	141	237	233	204	127	77	33	24
Allocated water to the Agriculture sector (MCM)	0	0	0	34	115	208	201	172	98	47	9	4.3
Allocated water to the other sectors* (MCM)	21.6	22.71	23.86	24.41	25.95	28.95	30.97	31.32	28.95	29.86	24.22	20
Average required demand for utility satisfaction equal one (MCM)	23	24	26	62	148	260	245	215	132	82	35	25

In order to evaluate the applicability of the optimal policy associated with water release (fourth row in the Table 3) a 30-year simulation model is run and the reliability indices for the reservoir manager and water users are calculated subsequently (Table 4).

**Table 4:** Volumetric reliabilities of the reservoir system resulting from the simulation based on the presented method.

Reliability $R_{v-all}^{T}{\;(R}_{v-st}^{T})$ *		Jan	Feb	Mar	Apr	May	Jun	Jul	Aug	Sep	Oct	Nov	Dec
Total reservoir system allocation indices		95.3	95.1	94.7	65.0	63.5	51.2	56.3	68.9	53.7	55.4	91.2	94.6
Reservoir operator total reservoir storage index) ${(R}_{v-st}^{T})$ *		0.0	0.0	0.0	0.005	0.024	0.047	0.016	0.018	0.045	0.019	0.011	0.002
Water users	Agriculture	100	100	100	62.3	61.58	46.48	70	66.95	45.37	48.5	93.4	97.5
	Other Users**	85.0	87.3	86.5	79.1	82.2	84.8	94.9	90.6	80.9	77.4	75.3	72.3

* ${(R}_{v-st}^{T})$: Total storage shortfall/ total available water during the planning horizon.

** Including: Domestic, Industrial and Environmental sectors are considered as one independent sector.

As can be seen in Table 4, the reliability values reduced by approximately forty percent over the first four months of the year for the agriculture sector. This was due to the considerable increase in water demand over this period. Although there is an average water demand of nearly 250 MCM in July, a considerable portion of the required water has been met.

### Solving the continuous dynamic model using collocation method

**Fig 4:**
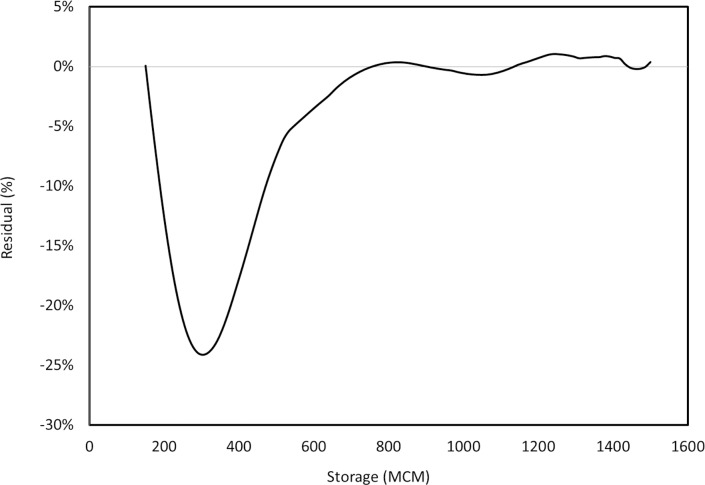
The residuals variation over the entire interpolation interval of the state variable for the Chebychev approximation function.

Regarding the curvature of the value function, the appropriate number of collocation nodes (dimensions of the problem) and the basis-node scheme were chosen. In this study, various dimensions and basis-node schemes were evaluated to render it computationally efficient. Ultimately, the Chebychev approximation function was employed to serve as the basic function while the appropriate dimension of the problem was considered to be 10 terms of corresponding basic functions. As the next step, the collocation equations were solved by applying Newton's solution method. Applying the Chebychev approximation function, the program converged in 372 iterations. Once the collocation method is used to cope with dynamic problems, the residual function is employed to evaluate the quality of the approximation. The residual function represents the difference between the left and right sides of this equation at arbitrary states *S*_*t*_. Fig 4 shows the changes in residual function over the range of the state variable for the considered approximation function. It appears that the applied approximation function enjoys an acceptable level of accuracy. There are some disturbances over the interval which can be due to the discontinuities of the derivatives of utility functions at those points.

**Fig 5:**
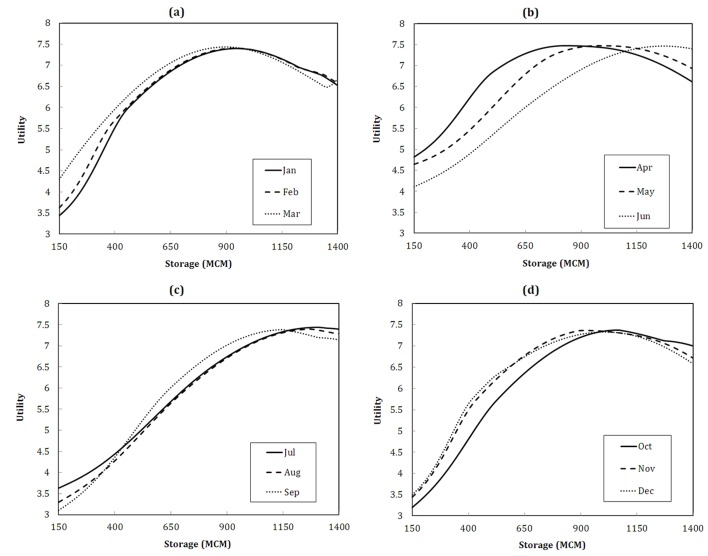
Value functions resulting from collocation solution. (a), the first three months, (b), the second three months, (c), the third three months and, (d), the fourth three months.

**Fig 6:**
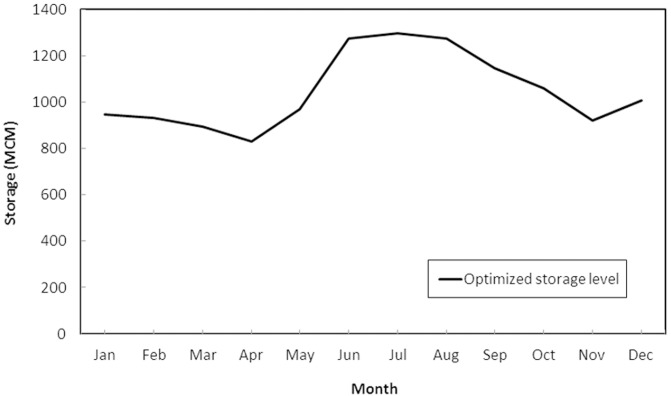
Optimal level of storage that results in the maximum long-term utility of the water users and reservoir operator in every month of the year.

Fig 5 represents the value functions of the solution provided for the applied scheme. Fig 6 illustrates the corresponding reservoir storages through which the maximum long-term utility of the water users and reservoir operator for each month is obtained. According to Fig 6, it is clear that over the summer season (from June to August) higher storage is required in the reservoir to obtain higher level of utility. Considering the high level of agricultural activity during this time of year, it is rendered appropriately.

**Fig 7:**
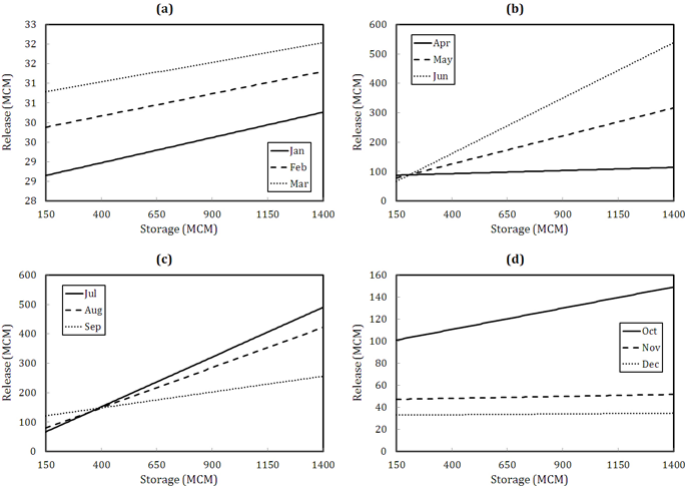
The operating policy resulting from the collocation solution, applying Chebychev approximation function.

Fig 7 illustrates the resulting policy function. It is obvious that, as long as the weather is moving toward the warm and dry season (July to September), the provided operating policy diagrams would go on a steeper gradient and consequently, it is logical that a higher level of release is obtained.

Considering the optimal reservoir operation rule, a simulation model based on a time period of 30 years is created to evaluate the optimal operating rule as well as the variation in water users' utilities. Subsequently, the reliability indices for reservoir manager and water users are calculated and presented in Table 5. Additional information associated with the simulation model and the optimization model including source code are available from authors upon the request.

**Table 5:** The results of simulation based on the outcomes of collocation method working on an annual basis done by Homayoun-far et al. [42].

Reliability		$R_{v-all}^{T}(R_{v-st}^{T})$ *	A.R.D (MCM)	A.S.D (MCM)	V.S.D (MCM)
Total reservoir system allocation indices		96.30	1277	1238.2	332
Reservoir operator (total reservoir storage index)		0.049	-	-	-
Water users	Agriculture	98.19	959.76	942.35	413
	Other Users**	94.95	316.92	315.93	67

* ${(R}_{v-st}^{T})$: Total storage shortfall/ total available water during the planning horizon.

**Other users: Domestic, Industrial and Environmental sectors are considered as one independent sector.

A.R.D. (U = 1): average required demand which sets utility satisfaction (US) equal to one.

A.S.D.: average supplied demand.

V.S.D.: variance of supplied demand.

### Drawing a comparison between presented models and other dynamic alternatives

According to the results, it seems the presented models successfully take the dynamics of the reservoir system into account and maximized long term utility of the system. They resulted in optimal reservoir operating policies and optimal water allocation in the reservoir system. Moreover, the state variable (water level in the reservoir) is considered as a continuous variable in the models thereby avoiding the dimensionality problem. There are, however, differences between them in term of the model structure. In the optimization model based on constrained state formulation (first model), the long-term variation of the state variable is imposed in the optimization model through constraints of the model. Therefore, the optimal value of long-term utilities corresponding to water users and reservoir management are achieved from the optimization. While, in the second model in order to maximize long-term utility value of the system, a form of the Bellman Equation is employed.

Considering the values of reliability that resulted from the 30-year simulation, it seems that both models can successfully deal with water conflict issues which may occur in reservoir management. In comparison with the reliabilities of the water users from the first model, the reliability values in the second model suggest higher performance in the management of water allocation. Considering the total reservoir storage index ${(R}_{v-st}^{T})$ also the second model produced more reliable values compared to the model based on the constrained state formulation. The higher performance of the second model in producing better reliability indices can be rooted in utilizing the structure of the Bellman Equation. Using this dynamic structure makes the modeling situation closer to the reality.

In comparison with the game model which works on an annual basis (Table 5), the second model results in better values (less values) of the total reservoir storage reliabilities (second row of Table 6). Regarding the water users, the reliabilities that resulted from the second model (fourth row of Table 6) precede the annual value. It should be noted that from the practical viewpoint, the results of models which work on a monthly basis, although providing lower reliabilities in a few cases, are more realistic when compared to annual methods.

**Table 6:** Volumetric reliabilities of the reservoir system resulting from the simulation based on the presented method.

Reliability $R_{v-all}^{T}{\;(R}_{v-st}^{T})$ *		Jan	Feb	Mar	Apr	May	Jun	Jul	Aug	Sep	Oct	Nov	Dec
Total reservoir system allocation indices		100.00	99.89	98.99	96.16	81.43	58.39	61.14	61.43	65.72	79.60	96.54	99.93
Reservoir operator (total reservoir storage index) ${(R}_{v-st}^{T})$ *		0.0000	0.0003	0.0004	0.0000	0.0000	0.0000	0.0000	0.0160	0.0529	0.1090	0.0337	0.0002
Water users	Agriculture	100.00	100.00	100.00	94.55	78.83	55.98	58.06	57.85	60.28	72.02	96.21	100.00
	Other Users**	100.00	99.89	98.92	100.00	100.00	99.62	99.76	100.00	98.36	97.82	96.70	99.92

* ${(R}_{v-st}^{T})$: Total storage shortfall/ total available water during the planning horizon

**Other users: Domestic, Industrial and Environmental sectors are considered as one independent sector

**Fig 8:**
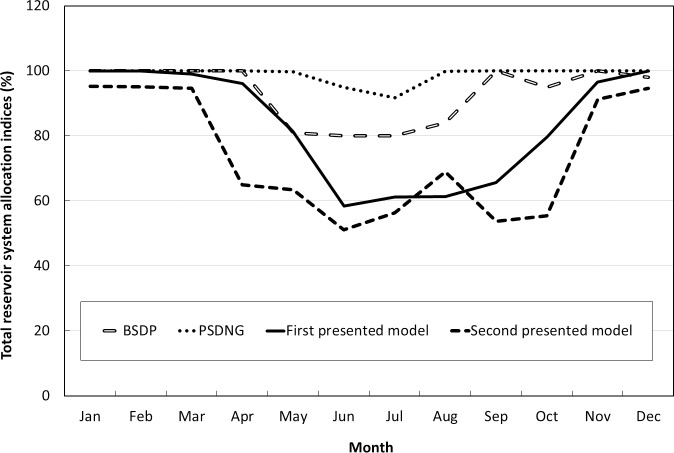
The volumetric reliability of the reservoir system associated with different models for Zayandeh-Rud river system.

In addition, the efficiency of the presented models can be further compared, using the information on the system reliability index of the BSDP model [45], PSDNG model [18], and an annual form of dynamic game [42] in Table 7. According to the results, in comparison with the BSDP and PSDNG, the presented models result in lower reliability values (Fig 8). Also, compared to the discrete dynamic games (i.e. PSDNG), runtime is considerably reduced. The discrete nature of the PSDNG model may be the main reason for the better results and longer runtime. The PSDNG is a stochastic conflict resolution model, which makes possible achievement of higher precision in optimization, using a fine discretization of state variables. Furthermore, the PSDNG model uses the Simulated Annealing (SA) procedure to search for the static equilibrium point in each state of n stages of the model. A fine discretization and using the SA procedure increases the runtime and causes dimensionality problems [18]. In addition, according to the results, when compared with the annual dynamic game models, the presented models provide superior results. Indeed, the operating policies resulting from the second model are more practical than those of the annual models [42, 44].

**Table 7:** The comparison of system reliability indices among presented models, annual form of dynamic game, PSDNG and BSDP model.

Reliability Index	Continuous dynamic optimization models			Discrete dynamic optimization models	
	Based on constrained state formulation (First presented model)	Based on continuous state Markov decision (Second presented model)	Annual dynamic game model*	PSDNG	BSDP
$R_{v-all}^{T}$	73.74	83.27	96.30	97.21	89.34
$R_{v-st}^{T}$	0.016	0.018	0.049	0.0	-
**A.R.D (MCM)**	1277	1277	1277	1277	1277
**A.S.D (MCM)**	1203	1234	1238.2	1228.8	1228.8

* Annual dynamic game model: presented by Homayounfar [42]

## Conclusion

In this study two monthly continuous dynamic models based on NBS were developed to tackle water allocation conflicts in a reservoir system. The first model, utilizing constrained state formulation, computed the first and second moments of the state variable and used them as constraints in maximizing long-term utility of the reservoir operators and water users. The second model was a monthly continuous dynamic game model which was solved by the collocation method. Applying this model, operating policies were generated for every month. Considering the optimal reservoir operation rule, a 30-year simulation model is developed and corresponding reliability indices for reservoir manager and water users were calculated. According to the results, it seems the continuous dynamic model solved by the collocation method (second model) provided superior results compared to the model based on the constrained state formulation. The volumetric reliabilities resulted from the second model are greater than the corresponding values associated with the first one along the year. This can be due to incorporating the Bellman Equation into model structure. Using this dynamic structure make the modeling situation closer to the reality.

Regarding the solution method of the second model, the collocation method was used to find a polynomial approximation to an unknown function and generate operating policies in the reservoir system. Meanwhile, the Chebychev basis-node scheme was employed as the basis function in the collocation method. The results showed that the collocation method and applied basis function are quite appropriate and accurate in approximating the functional equations and their derivatives.

In order to evaluate the efficiency of the presented models, the reliability values were compared with the information on the system reliability index of the BSDP model, the PSDNG model and an annual form of dynamic game. In this regard, the PSDNG and BSDP models resulted in higher level of volumetric reliability index in comparison with the corresponding values resulted from the presented models. This can be due to the discrete nature of the BSDP and PSDNG. In addition, the PSDNG model employs the Simulated Annealing (SA) procedure to achieve the static equilibrium point in each state. Although a fine discretization of state and decision variable and the SA can result in higher precision in the optimization process, it often increases runtime and causes dimensionality problems.

Regarding the results, the proposed models increased the overall storage reliabilities of the reservoir system compared to the annual alternative. In addition, volumetric reliabilities improved over the year. Also, in comparison with annual alternatives, the operating rules resulted from the presented models are more useful in practice. In term of applicability of the presented approaches, it shows that conflict on water consumption in a river basin under a reservoir system can be stated based on the monthly continuous dynamic approaches. Due to the continuous form of the variable, these approaches will not suffer from the dimensionality problems. It also makes possible consideration of uncertainty in input values (inflows) in reservoir operation. Incorporating the inflow uncertainty may not represent reality, but it is getting closer to real world conditions.

## Supporting Information

S1 Table Inflow data. (XLSX)

S2 Table Monthly value function. (XLSX)

S3 Table Monthly operating policies. (XLSX)
